# Pregnancy health and perinatal outcomes among Pacific Islander women in the United States and US Affiliated Pacific Islands: Protocol for a scoping review

**DOI:** 10.1371/journal.pone.0262010

**Published:** 2022-01-18

**Authors:** Rachel Suss, Madison Mahoney, Kendall J. Arslanian, Kate Nyhan, Nicola L. Hawley

**Affiliations:** 1 Yale College, Yale University, New Haven, CT, United States of America; 2 Department of Social and Behavioral Sciences, Yale School of Public Health, New Haven, CT, United States of America; 3 Harvey Cushing/John Hay Whitney Medical Library, Yale University, New Haven, CT, United States of America; 4 Department of Chronic Disease Epidemiology, Yale School of Public Health, New Haven, CT, United States of America; Emory University School of Public Health, UNITED STATES

## Abstract

This scoping review examines the literature on pregnancy and perinatal outcomes among Pacific Islander women in the United States (U.S.) and U.S.-affiliated Pacific Islands. Our aim was to identify research that disaggregated Pacific Islanders from other population groups. We conducted a systematic search of MEDLINE (Ovid), Embase (Ovid), CINAHL (EBSCO), and PsycINFO (Ovid) databases and a hand-search of grey literature. Forty-eight articles published between January 2010 and June 2020 were included. The majority of studies were conducted in Hawaii and utilized clinical record data. Infant outcomes were more commonly reported than maternal outcomes. We highlighted several limitations of the existing literature that included aggregation of Pacific Islanders with Asian American and other ethnic groups; limited comparison between Pacific Islander sub-groups; inadequate definitions of the nationality and ethnic composition of Pacific Islander groups; a lack of hypothesis-driven primary data collection and clinical trials; and underrepresentation of Pacific Islanders in population-based studies. Researchers should address these limitations to improve pregnancy and perinatal outcomes among Pacific Islanders, who comprise the second fastest growing ethnic minority in the U.S.

## Introduction

In recent years, the United States (U.S.) has garnered global attention for having the worst maternal health outcomes of any developed country. The only country whose maternal mortality rate is rising, the U.S. maternal mortality rate more than doubled from 10.3 per 100,000 live births in 1991 to 23.8 in 2014 [1, 2] and over two-thirds of the 700 deaths from pregnancy-related complications in the U.S. annually are avoidable [3]. Similarly, while the reported infant mortality rate in the U.S. has declined significantly over the past century, it remains higher than among its peer nations in Europe [4]. The higher burden of maternal and infant morbidity and mortality in the U.S. has been attributed to advancing maternal age at first pregnancy [5], higher prevalence of chronic pre-pregnancy health conditions, such as hypertension, diabetes, and obesity [6], an increasing cesarean section rate [7] and fragmentation of the health system (lack of coordination among different healthcare providers) [3, 8], which interrupts continuity of care.

Underlying the general U.S. trends are striking disparities by race. African-American women die from preventable pregnancy-related complications at three to four times the rate of Non-Hispanic White Women and are twice as likely to experience severe maternal morbidity (life-threatening pregnancy-related complications) [3, 9]. African-American women are also twice as likely to have an infant who dies by their first birthday [10]. Although disparities are greatest between African American and Non-Hispanic White Women, American Indian, Alaska Native, and Asian American women also have elevated pregnancy-related mortality and morbidity compared to Non-Hispanic White Women [11–14].

Pregnancy and perinatal outcomes among Pacific Islanders in the U.S. are particularly underreported. In 2016, more than 1.5 million residents of the U.S. and U.S. Affiliated Pacific Islands (USAPIs) reported having full or partial Pacific Islander ancestry, making Pacific Islanders the second fastest-growing U.S. minority group [15–17]. A greater burden of pre-pregnancy obesity-related cardiometabolic disease among Pacific Islanders than other racial/ethnic groups [18] likely raises the risk of poor pregnancy outcomes among this group. Additionally, there are significant social, economic, and political challenges facing some Pacific Islander groups, as a result of their varying residency status and access to U.S. social services [19], that may put pregnant women and their infants at additional risk.

One of the major challenges in understanding risk for poor pregnancy and perinatal outcomes among Pacific Islanders is the frequent aggregation of Pacific Islanders with Asian Americans for reporting purposes. Despite the fact that the Asian American/Pacific Islander population in the U.S. is made up of >50 different ethnic groups, prior to 1997 the small number of U.S. Pacific Islanders were aggregated with Asian Americans for census purposes (because of geographic proximity and efforts at the time by Asian American and Pacific Islander advocacy groups to align efforts to gain better representation). Although they are now able to report their ethnicity under specific Pacific Islander categories, data often continue to be aggregated in large national studies--both with Asian Americans and by combining Pacific Islander groups--because of issues of small sample size in each of the individual Pacific Islander groups. While there are some cultural ties and similarities across the various Pacific Islander groups, there are marked physiological differences/differences in disease risk and a small number of studies indicate that outcomes may vary by Pacific Islander sub-group within the larger racial category; for example, one study conducted in Hawaii noted differences in birth outcomes among Native Hawaiian, Micronesian, Samoan and ‘other Pacific Islander’ women [20].

To better characterize the health and pregnancy outcomes of Pacific Islander women, and to inform clinical and public policy decision making, the objective for this scoping review was to examine the existing literature on pregnancy and perinatal outcomes among Pacific Islander women in the U.S. and U.S.-affiliated Pacific Islands and identify potential gaps. Specifically, we aimed to identify research that offers outcomes for Pacific Islanders disaggregated from any other population group. Here we report findings of the scoping review including: (1) the pregnancy health and perinatal outcomes most commonly reported, (2) the Pacific Islander populations represented by the existing literature and their geographic distribution in the U.S., (3) the types of studies conducted, and (4) key study findings.

## Materials and methods

The scoping review was designed in accordance with the Preferred Reporting Items for Scoping Reviews (PRISMA-ScR) [21] and the protocol and search strategy were deposited on the Open Science Framework [22].

### Study population

Studies were limited to those conducted with Pacific Islanders living in the U.S. and USAPIs, which include American Samoa, Guam, the Commonwealth of the Northern Mariana Islands (CNMI), the Federated States of Micronesia (FSM) and the Republic of the Marshall Islands (RMI). Outcomes of people of Māori (the indigenous Polynesian people of New Zealand) ethnicity living in the U.S. were eligible for inclusion, yet people of New Zealand nationality, who do not identify as Māori, were not. Search terms related to Pacific Islander ethnicity were cross-checked with the U.S. Census categories and a recent, similar protocol by McElfish, et al. [23]. Studies were excluded if they reported outcomes from Pacific Islander populations outside of the U.S. or USAPIs or did not disaggregate Pacific Islanders from other racial/ethnic groups.

### Outcomes of interest

Eligible studies focused on pregnancy health, perinatal outcomes, or both. Pregnancy health outcomes refer to outcomes during the period of gestation, while perinatal outcomes were defined as outcomes during birth and in the 5 days immediately after. Outcomes were selected *a priori* and our search terms were based on those of other systematic reviews of maternal and perinatal health and by searching for synonyms of each condition. For reporting purposes, we describe maternal and infant outcomes separately. We excluded studies describing pregnancy and birth outcomes associated with the Zika virus, which briefly impacted USAPI communities between 2015-2016 [24, 25] since those outcomes were unlikely to be representative of general population trends.

### Search strategy

Our search strategy was developed using two concepts: Pacific Islanders and pregnancy and perinatal outcomes. Appropriate controlled vocabulary terms and keyword search terms were used (see **S1–S4 Tables**). To the extent allowed by bibliographic database indexing, articles that had non-U.S./USAPI geographic subject indexing were not retrieved; articles with a U.S./USAPI subject heading and articles with no geographic subject headings were retrieved. The search strategy was developed by a public health librarian (KN) in consultation with all coauthors and peer reviewed by an independent medical librarian.

#### Information sources

The following biomedical databases were searched for potentially relevant articles: MEDLINE (Ovid), Embase (Ovid), CINAHL (EBSCO), and PsycINFO (Ovid). Database searches took place in July (PsycInfo and Embase) and August (MEDLINE and CINAHL) 2020. In August 2020 the websites of two journals (the Pacific Journal of Reproductive Health and Pacific Health Dialog) were hand-searched because these journals publish highly relevant material and are not well indexed in major bibliographic databases, with a similar approach taken for data presented by national, state and territorial government agencies (including, for example, the Centers for Disease Control and Pacific Island Health Officers Association). Citation chaining was completed on all papers that met inclusion criteria.

#### Publication criteria

We included studies published in the English language between January 2010 and June 2020. Studies published in peer-reviewed journals and government reports were included in this review. Conference abstracts, and master’s theses were excluded but PhD dissertations were eligible for inclusion. Where review articles were generated by the search strategy, their references were reviewed for additional primary data sources, but the review articles themselves are not summarized in the results text.

### Data management

Search results were exported from bibliographic databases and deduplicated in EndNote by the Cushing/Whitney Medical Library Cross-Departmental team, using a documented method based on Bramer et al. [26]. The deduplicated results were imported to Covidence, an evidence synthesis web application. Any further duplicates identified by Covidence were reviewed manually to identify. Any relevant articles identified through hand-searching and citation chaining were also added to the Covidence project.

### Selection of sources and data extraction

Screening occurred in two stages (title/abstract and full text); at each stage, publications were reviewed by two authors independently. At the title/abstract screening stage, the process was piloted with 200 publications; four team members screened the titles included and met to discuss to reach consensus about how screening criteria were applied. As screening proceeded with two authors per text, reasons for exclusion were documented and any disagreements about inclusion/exclusion were resolved through discussion among all the authors. At least two authors extracted relevant information from the articles meeting eligibility criteria and compared their extractions. Data extraction focused on pregnancy and perinatal outcomes reported, data collection period and data source, setting and population, inclusion of Pacific Islanders, study design, major findings, and funding sources. Where comparisons were made between Pacific Islanders and other populations (for example, in reporting of prevalence estimates or relative risk) we reported comparisons with the primary reference group used by the original study authors. When extracting the major findings of studies we prioritized adjusted models (controlling for other variables), where possible, over descriptive statistics. Since the primary goal of the scoping review was to describe the state of the extant literature rather than formally synthesize its findings, we did not conduct quality assessments and included all studies regardless of potential biases in their design.

## Results

**Fig 1:**
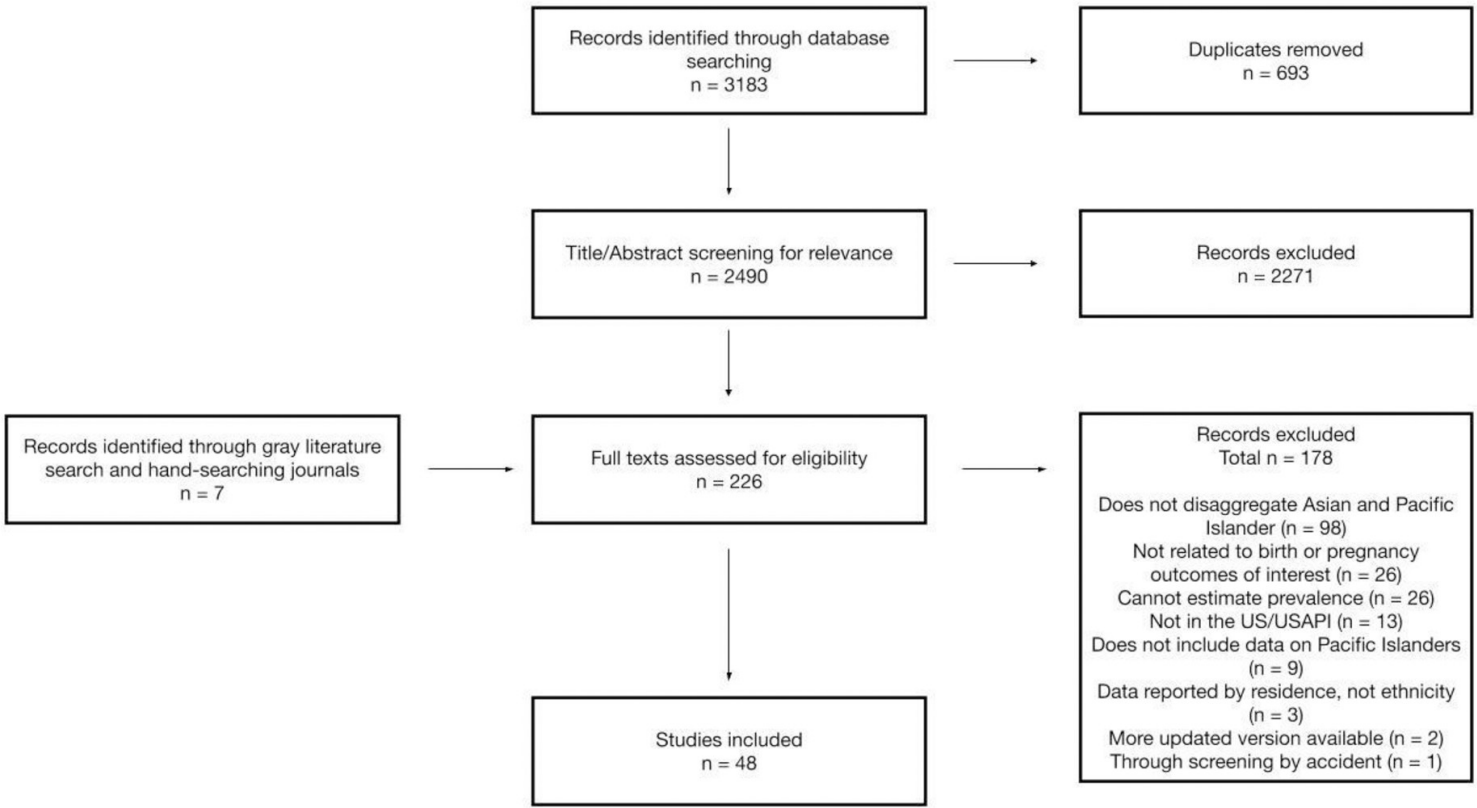
PRISMA flow diagram describing search outcomes.

Our initial search identified 2,490 unique records for which title/abstract screening was completed (**Fig 1**). Title/abstract screening removed 2,271 studies, which either did not include Pacific Islander populations or were not relevant to our outcomes of interest leaving 219 records for full text review. An additional 7 articles were identified through hand-searching of regional journals (n = 1) and the grey literature (n = 6). Of the 226 full texts assessed for eligibility, data extraction was completed for 48 studies, the results of which are summarized in **Tables 1** (Maternal Outcomes) and **2** (Infant Outcomes).

**Table 1:** Studies reporting maternal health outcomes among Pacific Islanders in the United States (US) and US-Affiliated Pacific Islands.

Author (year)	Data Collection	Setting/Population	Pacific Islanders (PI)	Results [Comparisons to the primary reference group used by study authors; OR/RR presented for adjusted models where available]
***Ectopic Pregnancy (n = 2 studies)***				
Stulberg et al., 2014 [27]	2000-2008	United States (n = 19,135,106 Medicaid recipients in 17 US states)	Native Hawaiian/PI (unspecified) (n not reported) Percentage of the PIs in the total sample could not be determined	• Native Hawaiian/PI vs. White: RR [95% CI] = 0.92 [0.90, 0.95]
Stulberg et al., 2016 [28]	2004-2008	United States (n = 19,135,106 Medicaid recipients in 17 US states)	Native Hawaiian/PI (unspecified) (n not reported) Percentage of the PIs in the total sample could not be determined	• Women of Native Hawaiian/PI ethnicity had a similar incidence of ectopic pregnancy (0.13% vs. 0.20%), but increased risk of complications following that ectopic pregnancy compared to White women (Incidence Risk Ratio (IRR) [95% CI] = 1.61 [1.39-1.87])
***Pregnancy Hypertension/Preeclampsia (n = 4 studies)***				
Nakagawa et al. 2016 [29]	1995-2013	Hawaii (n = 271,569 childbirth hospitalizations)	Native Hawaiian (n = 62,933) Other Pacific Islander (unspecified)(n = 22,245) PIs: 31.4% of total study sample)	• Outcome: Preeclampsia • Among non-obese women <35 years with singleton births: Native Hawaiian vs. White: OR [95% CI] = 1.54 [1.43, 1.66]; Other PI vs. White: OR [95% CI] = 1.40 [1.27, 1.54] • Among non-obese women ≥ 35 years with singleton births: Native Hawaiian vs. White: OR [95% CI] = 2.31 [2.00, 2.68]; Other PI vs. White: OR [95% CI] = 2.18 [1.79, 2.64] • Among obese women ≥ 35 years with singleton births: Native Hawaiian vs. White: OR [95% CI] = 1.80 [1.24, 2.60]; Other PI vs. White: OR [95% CI] = 1.68 [1.14, 2.49] • No significant differences observed between PI groups and White women when participants were obese, <35 years, with singleton births, or among women with multiple gestations
Singh et al., 2018 [30]	2014-2015	United States (n = 7,966,573 births recorded in national birth cohort data)	Hawaiian (n = 131,594) Samoan (n = 4,316) Percentage of the PIs in the total sample could not be determined due to some aggregation with Asians	• Outcome: Pregnancy-related hypertension • Hawaiian vs. Chinese: OR [95% CI] = 1.67 [1.35, 2.07]; Samoan vs. Chinese: OR [95% CI] = 1.66 [1.46, 1.89] • Outcome: Eclampsia • Prevalence of eclampsia was higher among Samoan (0.53%) and Hawaiian (0.52%) women than all other ethnic groups
Hawaii State Department of Health, 2019 [31]	2009-2015	Hawaii (n = 5,572 cross-sectional survey respondents)	Native/Part Hawaiian (n = 1,693) Samoan (n = 67) Guamanian (n = 17) Other PI (unspecified) (n = 253) PIs: 36.4% of total study sample	• 12.0% of Native/Part Hawaiian women, 23.3% of Samoan women, and 15.0% of Other PI women reported having high blood pressure, hypertension, preeclampsia, or toxemia during pregnancy compared to the state average of 11.0%
Lee et al., 2020 [32]	2008-2012	California (n = 2,518 childbirth hospitalizations among infants born between 500-1500g and 23-34 weeks gestation)	Pacific Islander (unspecified) (n = 124) PIs: 5.2% of total study sample	• 29.0% of Pacific Islanders had gestational hypertension compared to 31.6% of Asian women
***Gestational Diabetes (n = 9 studies)*** Studies comparing PIs (Hawaiian, Other PI, Samoan) to White women observed **greater prevalence** and **higher adjusted odds** of gestational diabetes among PI women compared to their White peers. Where Asian or Filipino women were the referent category, prevalence/odds were similar.				
Chang, Soon & Kaneshiro, 2010 [33]	1997-2006	Hawaii (n = 2,303 childbirth hospitalizations)	Micronesian (n = 2,303) PIs: 100% of total study sample	• 6.2% of Micronesian women were diagnosed with gestational diabetes during pregnancy
Hedderson, Darbinian & Ferrara, 2010 [34]	1995-2004	California (n = 216,089 births among the Kaiser Permanente Gestational Diabetes Registry)	Pacific Islander (unspecified) (n = 2,084) PIs: 1.1% of total study sample	• The age-adjusted prevalence of gestational diabetes was 7.2% (95% CI: 6.1, 8.3) among Pacific Islanders compared to 4.2% (95% CI: 4.1, 4.3) among non-Hispanic White women
Hunsberger et al., 2010 [35]	2004-2005	Oregon (n = 3,883 cross-sectional survey respondents with linked birth certificates)	Non-Hispanic Pacific Islanders (n = 76) Percentage of the PIs in the total sample could not be determined due to some aggregation with Non-Hispanic Asians	• 11.7% of PIs (9/76) had gestational diabetes (based on either self-report or listing on the birth certificate) compared to 6.0% of non-Hispanic White women • Small sample size prohibited calculation of odds ratios
Kim et al., 2013 [36]	2007-2009	California (n = 1,228,265 childbirth hospitalizations)	Pacific Islander (Guamanian, Hawaiian, Samoan, Other Pacific Islander) (*n* not reported) Percentage of the PIs in the total sample could not be determined due to some aggregation with Non-Hispanic Asians	• 10.5% (95% CI: 9.7-11.3%) of PIs had gestational diabetes compared to 5.4% of non-Hispanic White women
Tsai, Roberson & Dye, 2013 [37]	2009-2011	Hawaii (cross-sectional survey respondents with linked birth certificates) (*n* cannot be determined)	Hawaiian/Pacific Islander (Samoan, Guamanian, Other Pacific Islanders) (*n* cannot be determined)	• Hawaiian/Pacific Islander vs. White: unadjusted OR [95% CI] = 1.71 [1.19, 2.44]
Sugiyama et al., 2017 [38]	2007-2014	Palau (n = 1,730 childbirth hospitalizations)	Palauan (n = 1,449) PIs: 83.8% of total study sample	• Palauan vs. Other (Filipino, Other): OR [95% CI]: 0.96 [0.51, 1.81] • Models adjusted for maternal age and BMI
Hawaii State Department of Health, 2019 [31]	2009-2015	Hawaii (n = 5,572 cross-sectional survey respondents)	Native/Part Hawaiian (n = 1,693) Samoan (n = 67) Other Pacific Islander (Guamanian or Other) (n = 270) PIs: 36.4% of total study sample	• 10.4% of Native/Part Hawaiian women, 27.1% of Samoan women, and 16.0% of Other PI women reported being diagnosed with gestational diabetes during pregnancy compared to the state average of 12.4%
Martin et al., 2019 [39]	2018	United States (n = 3,791,712 records from US Natality data)	Native Hawaiian or Other Pacific Islander (unspecified) (n = 9,476) PIs: 0.25% of total study sample	• 8.6% of Native Hawaiian or Other PI women were diagnosed with gestational diabetes during pregnancy compared to 6.7% of women of all races/origins and 6.0% of non-Hispanic White women
Lee et al., 2020 [32]	2008-2012	California (n = 2,518 childbirth hospitalizations among infants born between 500-1500g and 23-34 weeks gestation)	Pacific Islander (unspecified) (n = 125) PIs: 5.0% of total study sample	• 18.6% of Pacific Islanders had gestational diabetes compared to 18.4% of Asian women
***Excess Gestational Weight Gain (n = 2 studies)***				
Chihara et al., 2014 [40]	2003-2005	Hawaii (n = 19,130 WIC recipients)	Hawaiian/Part Hawaiian (n = 6,780) Pacific Islander (unspecified) (n = 1,686) PIs: 44.2% of total study sample	• 67.8% of Hawaiian women and 69.3% of PI women exceeded the IOM gestational weight gain guidelines compared to 61.3% of White and 48.6% of Asian women
Hawley et al., 2015 [41]	2001-2008	American Samoa (n = 632 childbirth hospitalizations)	Samoan (n = 632) PIs: 100% of study sample	• 78% of Samoan women exceeded the IOM gestational weight gain guidelines
***Hepatitis B Virus (n = 2 studies)***				
Abara et al., 2018 [42]	2014	Guam (n = 966 prenatal care records)	PI (unspecified) (n = 752) PIs: 77.8% of total study sample	• 1.9% of PIs tested positive for Hepatitis B virus, compared to 2.2% of Asian women and 0.0% of White women
Noah, 2018 [43]	2014-2015	United States (n = 7,706,870 records from US Natality data)	Hawaiian (n = 1,701) Guamanian (n = 1,819) Samoan (n = 4,172) Other PI (unspecified) (n = 10,266) PIs: 0.23% of total study sample	• Hepatitis B virus was reported among 1.26% of PI women, with the other PI group (unspecified) experiencing higher prevalence (1.99%) compared to Hawaiian (0.18%), Guamanian (0.11%) and Samoan (0.43%) women. • PI vs. Asian Indian/Japanese American women: OR = 2.92 • Models adjusted for demographic characteristics, socioeconomic status, and pregnancy characteristics
***Cesarean/Assisted Birth (n = 10 studies) (Primary cesarean birth data is presented separately where available)*** Studies comparing PIs to White women that reported significant differences in the odds of cesarean/assisted birth **differed in their conclusions** depending on which PI group was included and what outcome was assessed. In general, cesarean births were similarly common among Hawaiian and non-Hispanic White women.				
Tsai et al., 2012 [44]	2009-2011	Hawaii (n = 200 primiparous women) Prospective cohort study	Hawaiian (n = 36) Micronesian (n = 31) PIs: 33.5% of total study sample	• Outcome: Assisted vaginal birth • 12.9% of Hawaiian and 15% of Micronesian women required assisted vaginal births, compared to 16.7% of White women
Sentell et al., 2014 [45]	2008-2012	Hawaii (n = 75,725 childbirth hospitalizations)	Native Hawaiian (n = 17,081) Other Pacific Islander (e.g. Samoan, Tongan, Micronesian) (n = 8,326) PIs: 33.6% of total study sample	• Outcome: Cesarean birth • Hawaiian vs. White: RR [95% CI] = 1.02 [0.98, 1.06]; Other PI vs. White: RR [95% CI] = 1.16 [1.10, 1.22] • Outcome: Primary cesarean birth • Hawaiian vs. White: RR [95% CI] = 0.83 [0.78, 0.88]; Other PI vs. White: RR [95% CI] = 1.16 [1.07, 1.26]
Chang et al., 2015 [20]	2010-2011	Hawaii (n = 15,156 childbirth hospitalizations)	Native Hawaiian (n = 6,662) Micronesian (n = 1,548) Samoan (n = 897) Other Pacific Islander (unspecified) (n = 539) PIs: 63.6% of total study sample	• Outcome: Cesarean birth • Native Hawaiian vs. White: OR [95% CI] = 0.93 [0.85, 1.01] • Micronesian vs. White: OR [95% CI] = 1.35 [1.19, 1.54] • Samoan vs. White: OR [95% CI] = 0.92 [0.77, 1.09] • Other Pacific Islander: OR [95% CI] = 1.31 [1.08, 1.59] • Models adjusted for age, rural residence, insurance, diabetes, hypertension and infant birth weight
Public Health Department, Seattle & King County, 2015 [46]	2013	Seattle (King County) (n = 24,910 births recorded in state data)	Native Hawaiian/Other Pacific Islander (unspecified) (n = 410) PIs: 1.64% of total study sample	• Outcome: Cesarean birth • 32% of births to Native Hawaiian or Other PI women were cesarean births, compared to the county average of 29% and 28% of births to non-Hispanic White women
Howells, Ah Ching, & Bender, 2016 [47]	2010-2011	American Samoa (n = 1,097 childbirth hospitalizations)	Samoan (n = 1,097) PIs: 100% of total study sample	• 22.5% of Samoan women experienced birth by cesarean section, 8.7% required an episiotomy, and 3% experienced an operative vaginal birth
Sentell et al., 2016 [48]	2012	Hawaii (n = 11, 419 childbirth hospitalizations)	Native Hawaiian (n = 2,506) Micronesian (n = 699) Other PI (unspecified)(n = 688) PIs: 34.1% of total study sample	• Outcome: Cesarean birth • Native Hawaiian vs. White: RR [95% CI] = 1.13 [0.84, 1.52] • Micronesian vs. White: RR [95% CI] = 1.54 [0.95, 2.48] • Other PI vs. White: RR [95% CI] = 0.87 [0.54, 1.39] • Models adjusted for maternal age, insurance status, rural vs. urban hospital location, and multiple gestation • Primary cesarean rate was not significantly different for any PI group compared to White women
Hawaii State Department of Health, 2019 [31]	2009-2015	Hawaii (n = 5,572 cross-sectional survey respondents)	Native/Part Hawaiian (n = 1,693) Samoan (n = 67) Other Pacific Islander (Guamanian or Other) (n = 270) PIs: 36.4% of total study sample	• Outcome: Cesarean birth • 22.9% of births to Native/Part Hawaiian women, 20.5% of births to Samoan women, and 27.2% of births to Other PI women were cesarean births compared to the state average of 24.4%
Martin et al., 2019 [39]	2018	United States (n = 3,791,712 records from US Natality data)	Native Hawaiian or Other Pacific Islander (unspecified) (n = 9,476) PIs: 0.25% of total study sample	• Outcome: Cesarean birth • 31.1% of births to Native Hawaiian or Other PI women were cesarean births, compared to 31.9% for all races and origins combined and 30.8% of births to non-Hispanic White women
Nembhard et al. 2019a [49]	1997-2013	Arkansas (n = 91,622, birth records, birth certificates)	Marshallese (n = 2,488) PIs: 2.7% of total study sample)	• Marshallese women were more likely to have a primary cesarean birth (PR = 1.13; 95% CI: 1.01, 1.27), forceps assisted birth (PR = 1.68; 95% CI: 1.16, 2.43), and vacuum-assisted birth (PR = 1.89; 95% CI: 1.60, 2.22) compared to non-Hispanic White women • Models adjusted for maternal age, education, parity, and marital status
Yamasato, Kimata & Burlingame, 2019 [50]	2008-2015	Hawaii (n = 25,594 childbirth hospitalizations)	Native Hawaiian/Other Pacific Islander (n = 7,768) PIs: 30.4% of total study sample	• 5.0% of Native Hawaiian or Other PI women required episiotomy during birth, compared to 8.1% of White women • Operative births (forceps or vacuum assisted) were required for 5.7% of NHOPI women, compared to 6.8% of White women.
***Labor Complications (n = 2 studies)***				
Harvey et al., 2017 [51]	1995-2013	Hawaii (n = 243,693 childbirth discharges)	Native Hawaiian/Other PI (n = 85,178) PIs: 35.0% of total study sample	• Outcome: Postpartum hemorrhage • Native Hawaiian/Other PI vs. White: OR [95% CI] = 1.40 [1.32, 1.48] • Models adjusted for type of birth, labor characteristics, gestational hypertension and diabetes, placental dysfunction, and obesity
Nembhard et al., 2019a [49]	1997-2013	Arkansas (n = 91,622, birth records, birth certificates)	Marshallese (n = 2,488) PIs: 2.7% of total study sample	• Outcome: Precipitous labor (lasting <2 hours) • Marshallese vs. Non-Hispanic White: PR [95% CI] = 2.65 [2.22, 3.17] • Models adjusted for maternal age, education, parity, and marital status • Outcome: Other complications of labor and delivery • Marshallese vs. Non-Hispanic White: PR [95% CI] = 1.67 [1.53, 1.82] • Models adjusted for maternal age, education, parity, and marital status
**Obstetric Trauma (n = 4 studies)**				
Tsai et al., 2012 [44]	2009-2011	Hawaii (n = 200 primiparous women) Prospective cohort study	Hawaiian (n = 36) Micronesian (n = 31) PIs: 33.5% of total study sample	• 9.7% of Hawaiian women, and 15.0% of Micronesian women experienced a severe laceration during birth compared to 16.7% of White women
de Silva et al., 2014 [52]	2002-2003	Hawaii (n = 1,842 childbirth hospitalizations)	Hawaiian/Part Hawaiian (n = 338) Micronesian (n = 56) Other PI (n = 57) PIs: 24.5% of total study sample	• Outcome: Severe laceration • Part Hawaiian/Hawaiian vs. Caucasian: OR [95% CI] = 0.78 [0.41, 1.50] • Micronesian vs. Caucasian: OR [95% CI] = 0.75 [0.26, 2.13] • Other PI vs. Caucasian: OR [95% CI] = 0.56 [0.17, 1.81] • Models adjusted for episiotomy and operative delivery
Sentell et al., 2014 [45]	2008-2012	Hawaii (n = 75,725 childbirth hospitalizations)	Native Hawaiian (n = 17,081) Other Pacific Islander (e.g. Samoan, Tongan, Micronesian) (n = 8,326) PIs: 33.6% of total study sample	• Outcome: Obstetric trauma during vaginal delivery without instrumentation • Hawaiian vs. White: RR [95% CI] = 0.62 [0.52, 0.74]; Other PI vs. White: RR [95% CI] = 1.09 [0.89, 1.34] • Outcome: Obstetric trauma during vaginal delivery with instrumentation • Hawaiian vs. White: RR [95% CI] = 0.66 [0.50, 0.87]; Other PI vs. White: RR [95% CI] = 0.80 [0.60, 1.06]
Yamasoto, Kimata & Burlingame, 2019 [50]	2008-2015	Hawaii (n = 25,595 childbirth hospitalizations for vaginal birth)	Native Hawaiian or Other PI (unspecified) (n = 7,768) PIs: 30.3% of total study sample	• Outcome: Obstetric anal sphincter injury • Native Hawaiian or Other PI vs. White: OR [95% CI] = 0.79 [0.62, 1.01] • Models adjusted for body mass index, birth weight, episiotomy, fetal head position, operative delivery, parity, and shoulder dystocia
***Maternal Mortality (during pregnancy or within one year postpartum) (n = 1 study)***				
Burlingame et al., 2012 [53]	1991-2007	Hawaii (n = 156 pregnancy-associated deaths)		• The pregnancy-related mortality ratio was 0.3 among those reporting Hawaiian ethnicity, 6.1 for those with part-Hawaiian ethnicity, and 4.2 for Other PI (unspecified) compared to 5.1 per 100,000 births among Caucasian women.

**Table 2:** Studies reporting infant health outcomes among Pacific Islanders in the United States (US) and US-Affiliated Pacific Islands.

Author (year)	Data Collection	Setting/Population	Pacific Islanders (PI)	Results [Comparisons to the primary reference group used by study authors; OR/RR presented for adjusted models where available]
***Preterm Birth (n = 18 studies) (Definition*: *birth prior to 37 weeks gestation unless otherwise specified)*** Studies comparing PIs (Hawaiian, Chamorro, Marshallese, Samoan, Guamanian, Tongan) to White women generally observed **greater prevalence** and **higher adjusted odds** of preterm birth among PI women compared to their White peers. Where Chinese women were the referent category, higher adjusted odds of preterm birth were also observed among Chamorro/Carolinian and Other PI women.				
Altman et al., 2019 [54]	2007-2012	California (n = 10,470 childbirth hospitalizations)	Hawaiian (n = 756) Guamanian (n = 844) Samoan (n = 2,852) Other PI (unspecified) (n = 5,422) More than one (n = 596) PIs: 100% of total study sample	• Overall rate of preterm birth was 8.3%; preterm birth was highest for women identifying as more than one PI group (9.9%) and lowest for women from Samoa or Guam (7.8%).
Hawaii State Department of Health, 2019 [31]	2009-2015	Hawaii (n = 5,572 cross-sectional survey respondents)	Native/Part Hawaiian (n = 1,693) Samoan (n = 67) Other PI (Guamanian or Other) (n = 270) PIs: 36.4% of total study sample	• 8.9% of infants born to Native/Part Hawaiian women, 7.1% of infants born to Samoan women, and 10.7% of infants born to Other PI women were preterm compared to the state average of 9.0%
Martin et al., 2019 [39]	2018	United States (n = 3,791,712 records from US Natality data)	Native Hawaiian or Other PI (unspecified) (n = 9,476) PIs: 0.25% of total study sample	• 8.57% infants born to Native Hawaiian or Other PI women were preterm, compared to 10.02% for all races and origins combined and 9.09% of non-Hispanic White women • 2.1% of infants born to Native Hawaiian or Other PI women were early preterm (born before 34 completed weeks of gestation); 6.46% were late preterm (34-36 weeks)
Nembhard et al., 2019a [49]	1997-2013	Arkansas (n = 91,622, birth records, birth certificates)	Marshallese (n = 2,488) PIs: 2.6% of total study sample)	• Marshallese vs. Non-Hispanic White: PR [95% CI] = 1.66 [1.50, 1.83] • Models adjusted for maternal age, education, parity, and marital status
Dela Cruz et al., 2018 [55]	2007-2014	Commonwealth of the Northern Mariana Islands (n = 8,427 childbirth hospitalizations)	Chamorro/Carolinian (n = 2,799) Other PI (unspecified) (n = 785) PIs: 42.5% of total study sample	• Chamorro/Carolinian vs. Chinese: OR [95% CI] = 2.7 [2.0, 3.6] • Other PI vs. Chinese: OR [95% CI] = 2.9 [2.1, 4.1] • Models adjusted for maternal age at delivery and number of antenatal care visits
Delara, Madden & Bryant, 2018 [56]	1999-2005	California (n = 189,931 childbirth hospitalizations among women with two pregnancies in the study period)	PI (unspecified) (n = 840) PIs: 0.4% of total study sample	• PI vs. White: OR [95% CI] = 1.51 [0.94, 2.40] • Models adjusted for maternal age, type of delivery, gestational age at first delivery, interpregnancy interval, maternal education, public insurance, nativity, prenatal care, multiple gestation, pre-existing diabetes mellitus, GDM, chronic and pregnancy hypertension
Ju et al., 2018 [57]	2000-2011	Hawaii (n = 20,061 cross-sectional survey respondents with linked birth certificate data)	Native Hawaiian or PI (n = 7,657) PIs: 38.1% of total study sample	• 9.1% (95% CI: 8.4-9.8%) of infants born to Native Hawaiian or PI women were preterm compared to 8.8% (95% CI: 8.2-9.3%) of infants born to non-Native Hawaiian or PI women (White, Filipino, Other Asian, Other)
Kim et al., 2018 [58]	2004	Hawaii (n = 17,677 records from US Natality Data)	Samoan (n = 544) Percentage of the PIs in the total sample could not be determined due to some aggregation with Asians, American Indians, and Alaska Natives	• 13.5% of infants born to US-born Samoan women and 10.0% of infants born to foreign-born Samoan women were preterm, compared to 8.0% and 6.9% of infants born to US- and foreign-born White women, respectively.
Ratnasiri et al., 2018 [59]	2007-2016	California (n = 435,280 preterm births; childbirth hospitalizations)	PI (Guamanian, Hawaiian, Samoan, Other PI) (*n* not reported) PIs: 8.6-10.8% of study sample depending on study year	• PI vs. White: OR [95% CI] = 1.43 [1.35, 1.51] • Models adjusted for birth year, maternal parity, maternal age, and education
Morisaki et al., 2017 [60]	2009-2012	United States (n = 10,638,415 records from US Natality Data)	Hawaiian (n = 152) Guamanian (n = 904) Samoan (n = 2,481) PIs: 0.03% of total study sample	• 9% of infants born to Hawaiian women, 13% of infants born to Guamanian women, and 12% of infants born to Samoan women were preterm compared to 8% of infants born to White women
Mattheus et al., 2016 [61]	2009-2011	Hawaii (n = 4,309 cross-sectional survey respondents)	Native Hawaiian/Part Hawaiian (n = 1,493) Other PI (unspecified) (n = 277) PIs: 41.0% of total study sample)	• Definition: Participant-reported signs and symptoms of labor more than 3 weeks before baby was due • Native Hawaiian/Part Hawaiian vs. White: OR [95% CI] = 1.73 [1.22, 2.46] • Other PI vs. White: OR [95% CI] = 0.52 [0.26-1.04] • Models adjusted for maternal age, education, marital status, insurance, county of residence, dental problems during pregnancy, oral hygiene counseling during pregnancy, smoking, alcohol, and prescription drug use during pregnancy, pre-pregnancy asthma, previous premature birth, and previous birth with normal birth weight
Sentell et al., 2016 [48]	2012	Hawaii (n = 11,419 childbirth hospitalizations)	Native Hawaiian (n = 2,506) Micronesian (n = 699) Other PI (unspecified) (n = 688) PIs: 34.1% of total study sample	• Native Hawaiian vs. White: RR [95%] = 1.25 [1.04, 1.51] • Micronesian vs. White: RR [95% CI] = 1.39 [1.07, 1.81] • Other PI vs. White: RR [95% CI] = 1.45 [1.13, 1.86] • Models adjusted for maternal age, insurance status, rural vs. urban hospital location, high risk pregnancy and multiple gestation • All PI groups were at greater risk of preterm birth than White women
Public Health Department, Seattle & King County, 2015 [46]	2013	Washington (King County) (n = 24,910 state birth records)	Native Hawaiian/Other PI (unspecified) (n = 410) PIs: 1.64% of total study sample	• 13.0% of infants born to Native Hawaiian/Other PI were preterm compared to the county average of 9.3%
Washington State Department of Health [62]	2015	Washington State (birth certificate data, n not reported)	PI (unspecified) (n not reported) Percentage of the PIs in the total sample could not be determined	• 14% of infants born to PI women were preterm compared to 7% of infants born to non-Hispanic White women
Hirai et al., 2013 [63]	2002-2009	Hawaii (n = 74,600 linked birth/infant death records)	Native Hawaiian (n = 40,917) PIs: 54.8% of total study sample	• Native Hawaiian vs. White: unadjusted PRR [95% CI] = 1.3 [1.3, 1.4]
Wong & Solet, 2011 [64]	2003-2008	Washington (King County) (n = 28,671 birth certificates)	Native Hawaiian and PI (Guam, Samoa, Fiji, Tonga, Micronesia, Melanesia, French Polynesia, Palau, Northern Marianas, Marshall Islands) (n = 2,442) PIs: 8.5% of total study sample	• 12.6% (95% CI: 11.1-14.2) of infants born to Native Hawaiian/PI women were preterm compared to 9.7% (95% CI: 9.3-10.1) of infants born to Asian women
Crowell et al., 2010 [65]	1995-2004	Hawaii (n = 177,955 State of Hawaii birth records)	Hawaiian/Part Hawaiian (n = 33,857) Samoan (n = 3470) PIs: 30.8% of total study sample	• 9.6% of infants born to Hawaiian/Part Hawaiian women and 7.9% of infants born to Samoan women were preterm compared to 7.7% of infants born to Caucasian women
Schempf et al., 2010 [66]	2003-2005	California and Hawaii (n = 647,835) birth certificates)	Native Hawaiian (n = 16,805) Guamanian (n = 1,406) Marshallese (n = 938) Samoan (n = 4,820) Tongan (n = 1,594) PIs: 3.9% of total study sample	• Native Hawaiian vs. White: OR [95% CI] = 1.25 [1.06, 1.48] • Guamanian vs. White: OR [95% CI] = 1.45 [1.16, 1.81] • Marshallese vs. White: OR [95% CI] = 2.10 [1.74, 2.55] • Samoan vs. White: OR [95% CI] = 1.41 [1.26, 1.58] • Tongan vs. White: OR [95% CI] = 1.31 [1.09, 1.56] • Models adjusted for maternal nativity, age, education, marital status, parity, prenatal care, tobacco use, and state of residence
***Low Birth Weight (<2,500g)/Small for Gestational Age (<10^th^ percentile) (n = 14 studies)*** Studies comparing PIs to White women generally observed **similar prevalence** and **similar adjusted odds** of low birth weight among PI women compared to their White peers. In the small number of studies that reported significantly different odds of low birth weight compared to White women, some PI groups (Marshallese, Native Hawaiian) had higher adjusted odds of low birth weight, while other groups (Samoans) had lower adjusted odds. When Filipino/Asian women were the referent category, odds/prevalence of low birth weight were significantly lower among PI women.				
Lee et al., 2020 [32]	2008-2012	California (n = 2,518 childbirth hospitalizations among infants born between 500-1500g and 23-34 weeks gestation)	PI (unspecified) (n = 125) PIs: 5.0% of total study sample	• Outcome: Small-for-gestational age • PI vs. Filipino: OR [95% CI] = 0.43 [0.22, 0.84] • Models adjusted for maternal age, maternal education, insurance type, multiparity, multiple births, and BMI
Martin et al., 2019 [39]	2018	United States (n = 3,791,712 records from US Natality data)	Native Hawaiian or Other PI (unspecified) (n = 9,476) PIs: 0.25% of total study sample	Outcome: Very Low Birth Weight (less than 1,500g) • 1.48% of infants born to Native Hawaiian or Other PI women were very low birth weight, compared to 1.38% for all races and origins combined and 1.02% of infants born to non-Hispanic White women • Outcome: Low Birth Weight • 8.97% of infants born to Native Hawaiian or Other PI women were low birth weight, compared to 8.28% for all races and origins combined and 6.91% of infants born to non-Hispanic White women
Nembhard et al., 2019a [49]	1997-2013	Arkansas (n = 91,622 birth records, birth certificates)	Marshallese (n = 2,488) PIs: 2.7% of total study sample)	• Outcome: Low Birth Weight • Marshallese vs. Non-Hispanic White: PR [95% CI] = 1.12 [0.96, 1.29] • Models adjusted for maternal age, education, parity, and marital status • Outcome: Small-for-Gestational Age • Marshallese vs. Non-Hispanic White: PR [95% CI] = 1.25 [1.12, 1.39] • Models adjusted for maternal age, education, parity, and marital status
Ju et al., 2018 [57]	2000-2011	Hawaii (n = 20,061 cross-sectional survey respondents with linked birth certificate data)	Native Hawaiian or PI (n = 7,657) PIs: 38.1% of total study sample	• Outcome: Low birth weight • 6.6% (95% CI: 6.0-7.1%) of infants born to Native Hawaiian or PI women were low birth weight compared to 7.1% (95% CI: 6.7-7.5%) of infants born to non-Native Hawaiian or PI women (White, Filipino, Other Asian, Other)
Kim et al., 2018 [58]	2004	Hawaii (n = 17,677 records from US Natality Data)	Samoan (n = 544) Percentage of the PIs in the total sample could not be determined due to some aggregation with Asians, American Indians, and Alaska Natives	• Outcome: Low birth weight • 5.8% of infants born to US-born Samoan women and 2.5% of infants born to foreign-born Samoan women were classified as having low birth weight, compared to 4.9% of infants born to US-born women and 4.6% of infants born to foreign-born White women.
Wartko, Wong & Enquobahrie, 2017 [67]	2008-2012	Washington (n = 113,760 childbirth hospitalizations)	Native Hawaiian/Other PI (Guamanian, Chamorro, Samoan) (n = 1,853) PIs: 1.6% of total study sample	• Outcome: Low birth weight • NHOPI vs. non-Hispanic White: OR [95% CI] = 0.88 [0.68, 1.14] • Models adjusted for infant sex, maternal age, preterm birth, gestational diabetes, pregnancy-induced hypertension, obesity
Sentell et al., 2016 [48]	2012	Hawaii (n = 11,419 childbirth hospitalizations)	Native Hawaiian (n = 2,506) Micronesian (n = 699) Other PI (unspecified)(n = 688) PIs: 34.1% of total study sample	• Outcome: Low birth weight • Native Hawaiian vs. White: RR [95%] = 1.15 [0.92, 1.45] • Micronesian vs. White: RR [95% CI] = 1.56 [1.11, 2.20] • Other PI vs. White: RR [95% CI] = 1.12 [0.81, 1.57] • Models adjusted for maternal age, insurance status, rural vs. urban hospital location, high risk pregnancy and multiple gestation
Chang et al., 2015 [20]	2010-2011	Hawaii (n = 15,156 childbirth hospitalizations)	Native Hawaiian (n = 6,662) Micronesian (n = 1,548) Samoan (n = 897) Other PI (unspecified) (n = 539) PIs: 63.6% of total study sample	• Outcome: Low birth weight • Native Hawaiian vs. White: OR [95% CI] = 1.16 [1.01, 1.34] • Micronesian vs. White: OR [95% CI] = 1.08 [0.87, 1.35] • Samoan vs. White: OR [95% CI] = 0.55 [0.40, 0.76] • Other PI: OR [95% CI] = 1.20 [0.88, 1.64] • Models adjusted for age, rural residence, insurance, diabetes and hypertension
Public Health Department, Seattle & King County, 2015 [46]	2013	Seattle (King County) (n = 24,910 births recorded in state data)	Native Hawaiian/Other PI (unspecified) (n = 410) PIs: 1.64% of total study sample	• Outcome: Very Low Birth Weight (less than 1,500g) • 0.7% of infants born to Native Hawaiian or Other PI women were very low birth weight, compared to the county average of 0.9% and 0.8% of infants born to non-Hispanic White women • Outcome: Low Birth Weight • 5.4% of infants born to Native Hawaiian or Other PI women were low birth weight, compared to the county average of 6.5% and 5.7% of infants born to non-Hispanic White women
Hawley et al., 2014 [68]	2001-2008	American Samoa (n = 795 childbirth hospitalizations)	Samoan (n = 795) PIs: 100% of total study sample	• Outcome: Low birth weight • Low birth weight was observed among 1.3% of infants
Xiang et al., 2014 [69]	1995-2010	Southern California (retrospective cohort of n = 29,544 women diagnosed with gestational diabetes mellitus)	PI (unspecified) (n = 142) PIs: 0.5% of total study sample	• Outcome: Small-for-gestational-age • PI vs. Non-Hispanic White: RR [95% CI] = 0.60 [0.31, 1.18] • Models adjusted for maternal age, education, insurance type, presence of comorbidities, pre-eclampsia/eclampsia and anti-hyperglycemic drug use
Hirai et al., 2013 [63]	2002-2009	Hawaii (n = 74,600 linked birth/infant death records)	Native Hawaiian (n = 40,917) PIs: 54.8% of total study sample	• Outcome: Low birth weight • Native Hawaiian vs. White: unadjusted PRR [95% CI] = 1.3 [1.3, 1.4]
Wong & Solet, 2011 [64]	2003-2008	Washington (King County) (n = 28,671 birth certificates)	Native Hawaiian and PI (Guam, Samoa, Fiji, Tonga, Micronesia, Melanesia, French Polynesia, Palau, Northern Marianas, Marshall Islands) (n = 2,442) PIs: 8.5% of total study sample	• Outcome: Very Low Birth Weight (less than 1,500g) • 1.3% (95% CI: 0.9-1.9) of infants born to Native Hawaiian/PI women were very low birth weight, compared to 1.0% (95% CI: 0.9-1.1) of infants born to Asian women • Outcome: Low Birth Weight • 5.5% (95% CI: 4.6-6.6) of infants born to Native Hawaiian/PI women were low birth weight, compared to 7.5% (95% CI: 7.2-7.9) of infants born to Asian women
Schempf et al., 2010 [66]	2003-2005	California and Hawaii (n = 647,835) birth certificates)	Native Hawaiian (n = 16,805) Guamanian (n = 1,406) Marshallese (n = 938) Samoan (n = 4,820) Tongan (n = 1,594) PIs: 3.9% of total study sample	• Outcome: Low birth weight • Native Hawaiian vs. White: OR [95% CI] = 1.09 [0.87, 1.37] • Guamanian vs. White: OR [95% CI] = 1.30 [0.97, 1.75] • Marshallese vs. White: OR [95% CI] = 1.38 [1.06, 1.81] • Samoan vs. White: OR [95% CI] = 0.85 [0.71, 1.01] • Tongan vs. White: OR [95% CI] = 1.09 [0.85, 1.40] • Models adjusted for maternal nativity, age, education, marital status, parity, prenatal care, tobacco use, and state of residence
***Macrosomia (≥4000g)/Large for Gestational Age (>90^th^ percentile) (n = 8 studies)*** Studies comparing PIs to White women that reported significant differences in the odds of macrosomia or large-for-gestational age **differed in their conclusions** depending on which PI group was included and what outcome was assessed.				
Nembhard et al., 2019a [49]	1997-2013	Arkansas (n = 91,622, birth records, birth certificates)	Marshallese (n = 2,488) PIs: 2.7% of total study sample	• Outcome: Macrosomia • Marshallese vs. Non-Hispanic White: PR [95% CI] = 0.43 [0.35, 0.53] • Models adjusted for maternal age, education, parity, and marital status • Outcome: Large-for-Gestational Age • Marshallese vs. Non-Hispanic White: PR [95% CI] = 0.59 [0.50, 0.70] • Models adjusted for maternal age, education, parity, and marital status
Dela Cruz et al., 2018 [55]	2007-2014	Commonwealth of the Northern Mariana Islands (n = 8,427 childbirth hospitalizations)	Chamorro/Carolinian (n = 2,799) Other PI (unspecified) (n = 785) PIs: 42.5% of total study sample	• Outcome: Macrosomia • Chamorro/Carolinian vs. Filipino: OR [95% CI] = 2.4 [1.7, 3.5] • Other PI vs. Filipino: OR [95% CI] = 2.3 [1.4, 3.6] • Models adjusted for maternal age at delivery and number of antenatal care visits
Ju et al., 2018 [57]	2000-2011	Hawaii (n = 20,061 cross-sectional survey respondents with linked birth certificate data)	Native Hawaiian or PI (n = 7,657) PIs: 38.1% of total study sample	• Outcome: Macrosomia • 8.9% (95% CI: 8.1-9.7%) of infants born to Native Hawaiian or PI women had macrosomia compared to 7.5% (95% CI: 7.0-8.1%) of infants born to non-Native Hawaiian or PI women (White, Filipino, Other Asian, Other)
Sentell et al., 2016 [48]	2012	Hawaii (n = 11,419 childbirth hospitalizations)	Native Hawaiian (n = 2,506) Micronesian (n = 699) Other PI (unspecified) (n = 688) PIs: 34.1% of total study sample	• Outcome: Macrosomia • Native Hawaiian vs. White: RR [95%] = 0.89 [0.73, 1.09] • Micronesian vs. White: RR [95% CI] = 0.97 [0.66, 1.42] • Other PI vs. White: RR [95% CI] = 1.73 [1.38, 2.18] • Models adjusted for maternal age, insurance status, rural vs. urban hospital location, high risk pregnancy and multiple gestation
Chang et al., 2015 [20]	2010-2011	Hawaii (n = 15,156 childbirth hospitalizations)	Native Hawaiian (n = 6,662) Micronesian (n = 1,548) Samoan (n = 897) Other PI (unspecified) (n = 539) PIs: 63.6% of total study sample	• Outcome: Macrosomia • Native Hawaiian vs. White: OR [95% CI] = 0.84 [0.74, 0.96] • Micronesian vs. White: OR [95% CI] = 0.67 [0.53, 0.86] • Samoan vs. White: OR [95% CI] = 1.87 [1.51, 2.32] • Other PI vs White: OR [95% CI] = 1.23 [0.93, 1.64] • Models adjusted for age, rural residence, insurance, diabetes and hypertension
Hawley et al., 2015 [41]	2001-2008	American Samoa (n = 632 childbirth hospitalizations)	Samoan (n = 632) PIs: 100% of study sample	• Outcome: Large-for-Gestational Age • Large-for-gestational age was observed among 15.5% of infants in the cohort
Hawley et al., 2014 [68]	2001-2008	American Samoa (n = 795 childbirth hospitalizations)	Samoan (n = 795) PIs: 100% of total study sample	• Outcome: Macrosomia • Macrosomia was observed among 20.6% of infants
Xiang et al., 2014 [69]	1995-2010	Southern California (retrospective cohort of n = 29,544 women diagnosed with gestational diabetes mellitus)	PI (unspecified) (n = 142) PIs: 0.5% of total study sample	• Outcome: Large-for-Gestational Age • PI vs. Non-Hispanic White: RR [95% CI] = 1.21 [0.83, 1.76] • Models adjusted for maternal age, education, insurance type, presence of comorbidities, pre-eclampsia/eclampsia and anti-hyperglycemic drug use
***Birth Injury/Trauma to Neonate (n = 3 studies)***				
Nembhard et al., 2019a [49]	1997-2013	Arkansas (n = 91,622, birth records, birth certificates)	Marshallese (n = 2,488) PIs: 2.7% of total study sample	• Outcome: Birth injury • Marshallese vs. Non-Hispanic White: PR [95% CI] = 2.13 [1.50, 3.03] • Models adjusted for maternal age, education, parity, and marital status
Yamasoto, Kimata & Burlingame, 2019 [50]	2008-2015	Hawaii (n = 25,594 childbirth hospitalizations)	Native Hawaiian/Other PI (unspecified) (n = 7,768) Percentage of the PIs in the total sample could not be determined due to aggregation of a multiracial group	• Outcome: Shoulder dystocia • 1.4% of infants born to women of Native Hawaiian/Other PI ethnicity had shoulder • dystocia compared to 1.3% of infants born to White women
Sentell et al., 2016 [48]	2012	Hawaii (n = 11,419 childbirth hospitalizations)	Native Hawaiian (n = 2,506) Micronesian (n = 699) Other PI (unspecified)(n = 688) PIs: 34.1% of total study sample	• Outcome: Birth trauma- Injury to Neonate • Native Hawaiian vs. White: RR [95% CI] = 0.69 [0.22, 2.16] • Micronesian vs. White: RR [95% CI] = 1.88 [0.33, 10.80] • Other PI vs. White: RR [95% CI] = 0.92 [0.18, 4.63] • Models adjusted for maternal age, insurance status, rural vs. urban hospital location, high risk pregnancy and multiple gestation
***Birth Defects (n = 2 studies)***				
Nembhard et al., 2019b [70]	1997-2013	Arkansas (n = 91,622, birth records, birth certificates)	Marshallese (n = 2,488) PIs: 2.7% of total study sample	• Outcome: All birth defects • Marshallese vs. Non-Hispanic White: PR [95% CI] = 0.7 [0.5, 0.9] • Models adjusted for maternal age, education, parity, and marital status • For some specific abnormalities (ophthalmic and cardiovascular, prevalence was significantly higher among Marshallese than Non-Hispanic White women)
Rocha, Zalud & Dye, 2014 [71]	2006-2010	United States (n = 20,773,296 records from US Natality Data)	Hawaiian/Part Hawaiian (n = 5,918) Samoan (n = 8,905) Guamanian (n = 6,417) Percentage of the PIs in the total sample could not be determined due to some aggregation with Asians	• Outcome: Abdominal Wall Defects/10,000 births • Hawaiian: 8.45 [95% CI: 3.61, 19.76] • Samoan: 5.61 [95% CI: 2.40, 13.14] • Guamanian: 4.68 [95% CI: 0.48, 15.38]
***Other Birth Outcomes (n = 2 studies)***				
Nembhard et al., 2019a [49]	1997-2013	Arkansas (n = 91,622, birth records, birth certificates)	Marshallese (n = 2,488) PIs: 2.7% of total study sample	• Outcome: Infant anemia • Marshallese vs. Non-Hispanic White: PR [95% CI] = 3.10 [2.01, 4.77] • Models adjusted for maternal age, education, parity, and marital status • Outcome: Require assisted ventilation <30 minutes • Marshallese vs. Non-Hispanic White: PR [95% CI] = 2.11 [1.64, 2.71] • Models adjusted for maternal age, education, parity, and marital status
Wolforth, Loo & Sood, 2016 [72]	1996-2006	Hawaii (n = 1,525 infants born ≤32 weeks)	Native Hawaiian/Other PI (Hawaiian, Part Hawaiian, PI, Other PI, Marshallese, and Samoan) (n = 521) PIs: 34.2% of total study sample	• Outcome: Severe Retinopathy of Prematurity • Hawaiian/Other PI vs. all other ethnicities: OR [95% CI] = 0.38 [0.20, 0.73] • Models adjusted for gestational age, birth weight, receipt of postnatal steroids, and necrotizing enterocolitis
***Neonatal Mortality Rate/1*,*000 births (0-27 days after birth) (n = 1 study)***				
Ely & Driscoll, 2019 [73]	2017	United States (n = 22,341 linked infant death/birth records)	Native Hawaiian/Other PI (unspecified) (n not reported) Percentage of the PIs in the total sample could not be determined	• Native Hawaiian or Other PI = 3.82
Hirai et al., 2013 [63]	2002-2009	Hawaii (n = 74,600 linked birth/infant death records)	Native Hawaiian (n = 40,917) PIs: 54.8% of total study sample	• Native Hawaiian = 5.0 vs. White = 2.8 • Native Hawaiian vs. White: unadjusted RR [95% CI] = 1.8 [1.4, 2.3]

The primary reason for exclusion of papers at the full text stage is presented in **Fig 1,** although papers that were excluded may have been excluded for more than one of these reasons. Aggregation of Pacific Islanders with Asian Americans was the most common reason for excluding studies at the full text stage (n = 98 records). Other common reasons for exclusion were lack of data on our outcomes of interest (n = 26 records) and lack of data to estimate outcome prevalence (n = 25 records). Where disaggregated data on Pacific Islanders was presented we found the definition of ‘Pacific Islander’ to be lacking in consistency or to be frequently unreported (n = 29 of our 48 extracted studies either did not report the ethnicity/nationality of their Pacific Islander group at all or included an ‘Other Pacific Islander’ group). Some of the populations with greater representation in the U.S. e.g. Hawaiians, Guamanians, and Samoans, were reported as individual groups, while other, smaller populations such as Tongans or Micronesians were grouped into “Other Pacific Islander” categories whose composition was again, rarely defined, but in several cases made up the largest reporting group (for example, Noah et al., 2018 [43]). Often, the included Pacific Islander group made up only a small proportion of the total study sample. In studies that utilized U.S. national-level data, in particular, the proportion of Pacific Islanders in the study sample ranged from 0.03-0.25% [39, 43, 61].

**Fig 2:**
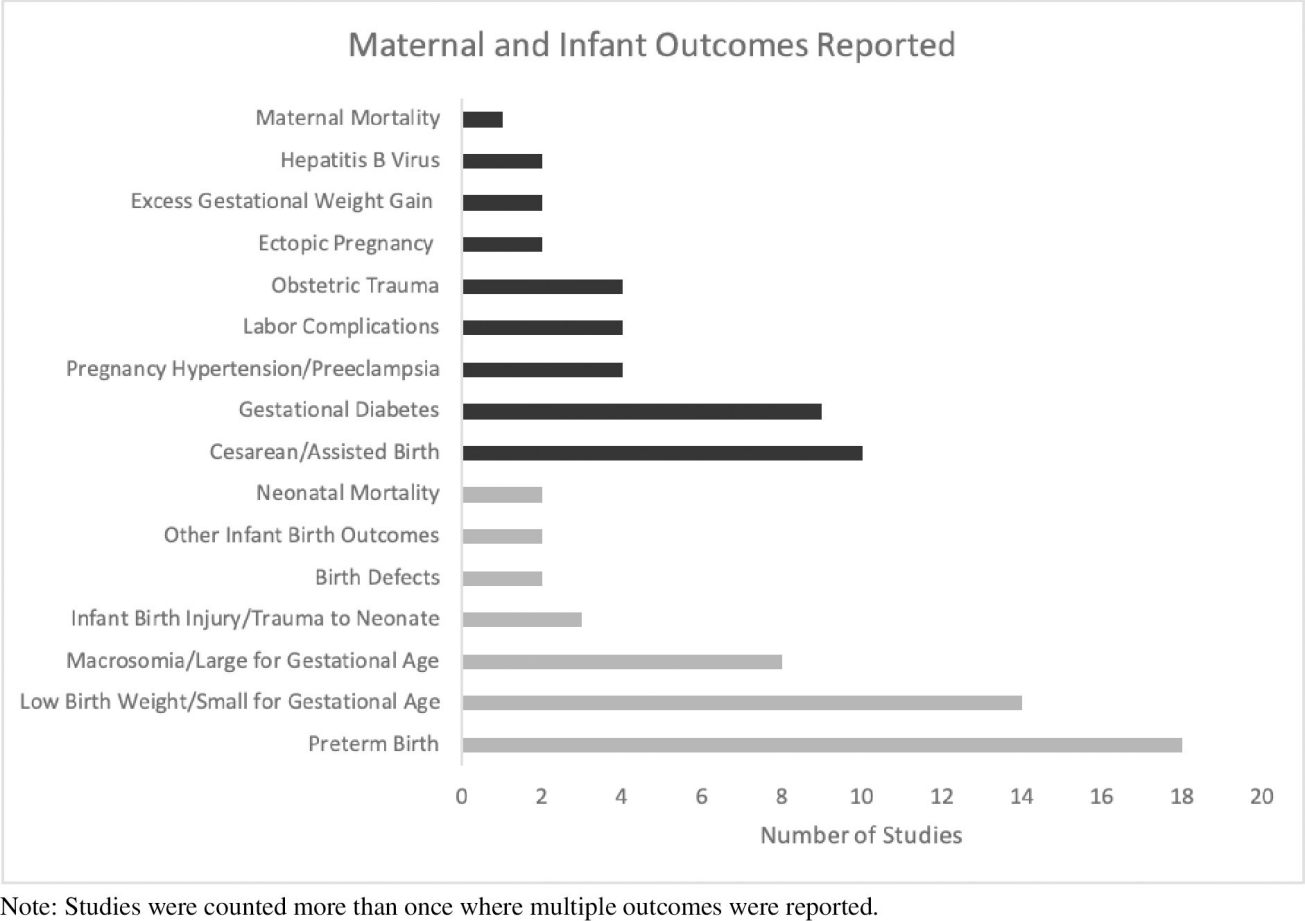
Number of studies that reported each maternal and infant outcome for Pacific Islanders.

**Fig 2** summarizes the maternal and infant outcomes reported by the 48 studies included in **Tables 1 and 2**; studies were counted separately for each outcome reported. The most commonly reported maternal outcomes were cesarean/assisted birth (n = 10 studies) and gestational diabetes (n = 9 studies), while the most commonly reported infant outcomes were preterm birth (n = 18 studies) and low birth weight/small-for-gestational age (n = 14 studies). Maternal and neonatal mortality were the least commonly reported outcomes.

Of the extracted studies, n = 9 utilized either U.S. national-level data (e.g. U.S. Natality or Medicare data) or data collected from multiple states. The vast majority of studies (n = 21) used data collected in Hawaii and Hawaiians were the most commonly represented population group. Studies presenting data from other U.S. states were conducted in California (n = 8), Washington (n = 4), Arkansas (n = 2), and Oregon (n = 1), while relatively fewer utilized data from the U.S.-affiliated Pacific Islands (n = 6 total studies). While our inclusion criteria specified that papers must have been published between 2010 and early 2020, 35% of the included papers (n = 17/48) reported on data collected prior to 2010, and only n = 8 of the 48 extracted papers reported on data collected in 2015 or after [30, 31, 39, 43, 50, 60, 63, 66].

The vast majority of studies were cross-sectional in design, descriptive in nature, and all were based on secondary analyses of clinical record data associated with childbirth hospitalizations. We identified no case-control or randomized controlled trials where Pacific Islander women and their pregnancy/birth outcomes were the primary focus nor any primary data collection with prospective follow up occurring within these groups. Where comparisons between the outcomes of Pacific Islander women/infants and those from other ethnic groups were made, the majority of studies (31 of 39) compared Pacific Islanders to non-Hispanic White women. While it was not an explicit objective of this scoping review, **Tables 1 and 2** offer brief summaries of the aggregate findings where there were more than n = 5 studies on a given outcome of interest. In line with the secondary nature of the analyses conducted across the included studies, few authors reported having received funding for the primary purpose of supporting their analyses: 20 studies reported either receiving no funding or did not provide a disclosure; 21 studies reported grant funding from the U.S. National Institutes of Health, Centers for Disease Control, or the Agency for Healthcare Research and Quality but in all but five cases the funding supported authors/infrastructure, rather than being awarded specifically to address the research questions presented.

## Discussion

This scoping review examined the existing literature on the pregnancy and perinatal health of Pacific Islander women and their infants in the U.S. Our findings highlight several limitations of the existing literature, including continued aggregation of Pacific Islanders with Asian Americans and other ethnic groups, little comparison between Pacific Islander sub-groups, lack of attention to defining the nationality/ethnic composition of Pacific Islander groups, a lack of hypothesis-driven primary data collection and clinical trials, and underrepresentation in population-based studies.

The continued aggregation of Asian Americans and Pacific Islanders presents an ongoing challenge for researchers and policymakers – doing so prevents complete understanding of how race and ethnicity may be related to health outcomes and limits targeting of specific prevention or intervention efforts for these groups during the prenatal and perinatal periods. Data describing socio-demographic and economic differences between these two groups consistently demonstrates how aggregation obscures the specific needs of either group; for example data from the American Community Survey (2011-2015) reports that 17% of Asian American/Pacific Islanders are below the federal poverty line, when in fact this applies to only 13% of Asian Americans and 21% of Pacific Islanders [74]. Differences in outcomes for these two groups have been replicated for a number of other vital statistics (e.g. deaths), adverse health outcomes, and health risk behaviors [75–78]. One paper included in our final review, which described maternal, pregnancy, and birth characteristics of Asian and Native Hawaiian/Pacific Islander women in King County, Washington showed that Native Hawaiian and Pacific Islander women were more likely to be overweight or have obesity before pregnancy, smoke during pregnancy, or become pregnant as adolescents, highlighting how social and clinical services may need to be targeted differently to these groups [65]. Beyond the issue of aggregation with Asian Americans, we found that relatively few papers included in our review offered separate outcomes for Pacific Islander sub-groups (i.e. Hawaiian vs. Samoan vs. Fijian, etc.) or even reported the composition of their Pacific Islander groups. While there is a high degree of shared population ancestry among the Pacific Islander groups [79], outcomes associated with other health conditions support the need for further disaggregation of this group. Polynesians, for example, tend to be more susceptible to obesity – and therefore may carry additional risk into pregnancy – compared to either Micronesians or Melanesians [80, 81].

Several studies acknowledged that disaggregating Pacific Islander ethnicities into their individual subgroups would have strengthened their analyses, but in many cases, they cited small sample size as a barrier to doing so [35, 43, 50, 58, 67, 69]. Despite the fact that 0.4% of the U.S. population identified as Pacific Islander in the 2020 census [82], and that this group grew by 35% from 2000 to 2010 [16] (more than three times faster than the rate of the overall U.S. population) and another 28% between 2010 and 2020 [82], representation – particularly in studies using national-level data – fell short of what may have been expected. This may have occurred either as a result of several studies excluding those who identified as multiple races (which is common among Pacific Islanders) or because the studies included utilized clinical record data. Marshallese, Native Hawaiian, Samoan, and Chamorro populations have been shown to have limited access to healthcare benefits in the U.S. and are less likely to have private insurance or have ever received a pneumococcal vaccination, compared with all U.S. adults [83, 84]. Further, the number of home births has increased in the last decade in the U.S., reaching 0.54% in 2012 for Asian or Pacific Islander women [85], possibly contributing to the lack of data due to fewer hospital birth records. Understanding why this underrepresentation occurred may give additional clues to social service and public health needs of Pacific Islanders.

Geographically, the studies we included in our review represented the states with the largest proportion of Native Hawaiian and Pacific Islanders: Hawaii, California, Washington, Arkansas, and Oregon [16]. Studies completed in Hawaii disaggregated Pacific Islander subgroups more commonly than those completed elsewhere, likely because of the importance of documenting Native Hawaiian health outcomes (and therefore disaggregating those data from other Pacific Islanders), as well as the larger number of Pacific Islander women who could be included in analysis. This could also be a reflection of the recognition, knowledge, and value that Hawaiian researchers, and the society at large, have for differences in Pacific Islander ethnicities and cultures, compared with the mainland United States [86]. Less well represented were the U.S. territories - American Samoa, Guam, and the Commonwealth of the Northern Mariana Islands (CNMI) - and the three independent nations in free association with the U.S.: the Federated States of Micronesia (FSM) and the Republic of the Marshall Islands (RMI), and the Republic of Palau. Together these represent a population of more than 500,000 whose health needs, given their considerable reliance on the U.S. for health infrastructure and funding, require further investigation and documentation [87]. It should be noted that we excluded two studies conducted during the full text review stage when it was determined that those studies reported outcomes among women giving birth, for example, at the national hospital in Guam but did not explicitly state the ethnicity of the women included (Guam has a considerable expatriate population including non-Hispanic White and Chinese women).

Finally, among the 48 studies we identified, the most commonly reported outcomes were for infants, with relatively less attention paid to either maternal pregnancy health or associated birth outcomes. Given the source of the data, this may be unsurprising since outcomes such as birth weight and gestational age are routinely recorded in clinical records and can be accessed through a number of publicly available data repositories. Recording maternal health during pregnancy and, further, distinguishing between pre-existing and pregnancy-related conditions is relatively more challenging. Because Pacific Islanders live with higher rates of obesity compared to other ethnic and racial groups [80], we expected to see a greater number of studies focused on related pregnancy comorbidities (gestational hypertension or diabetes) or explicitly testing the association of obesity with pregnancy/perinatal outcomes. Although pregnancy hypertension, and gestational diabetes were represented among the 48 studies we identified here, a total of 13 papers (including also those focused on excess gestational weight gain) written on these obesity-related comorbidities over the last 10 years leaves a critical gap in the clinical literature for this group. Notably, several studies of pregnancy hypertension and diabetes had to be excluded for not distinguishing between pre-existing and pregnancy-induced dysfunction.[20, 88] While the main goal of this paper was not to compare Pacific Islanders’ risk for poor pregnancy/perinatal outcomes to those of other groups, but rather to identify gaps in the research, it should be noted that the majority of studies found that Pacific Islanders had higher prevalence, risk, or odds of having adverse outcomes compared to their white counterparts [27–32, 34–39, 41–49, 51, 52, 58–65, 67–73]. The underlying cause of these disparities needs further research but may be due to lack of healthcare access, discrimination, or other social determinants as have been reported among other U.S. minority/immigrant populations [89, 90].

This study has several strengths including its identification of issues with current reporting practices needed to improve the health of U.S. Pacific Islanders, our use of the gold-standard PRISMA scoping review method [21], and our thoroughness and attention to consistency throughout screening. All authors pretested the screening forms and conducted revisions as necessary before we extracted data. This analysis may also have some limitations. First, we may not have identified all data on Pacific Islander pregnancy and perinatal health from the gray literature - despite our aim to be as exhaustive as possible - since this search was done by targeting well-known agencies and state departments, which may have left out smaller, less accessible data sources. Our search was also performed using English terms and only included literature written in English, which may have precluded some data reported in other languages. Finally, we acknowledge as with all reviews, that the screening and extraction processes were subject to biases of the reviewers.

In conclusion, we recommend that maternal-child health researchers design, and relevant national organizations advocate for, studies that disaggregate Pacific Islander groups to ensure the health needs of these communities are both documented and met. Researchers can achieve this by utilizing databases with sufficient sample size of Pacific Islander ethnicities and by prioritizing movement from descriptive studies to case-control or randomized controlled study designs, where Pacific Islander women and their pregnancy/birth outcomes are the primary focus. The 1.6 million + Pacific Islanders who live in the mainland U.S. and the USAPIs [91] have a right to evidence-based care - beginning with clinical studies that disaggregate these groups - to advance health equity.

## Supporting information

S1 Checklist (DOCX)

S1 Table Ovid/MEDLINE search strategy for studies related to pregnancy and perinatal health outcomes among Pacific Islander women in the United States and U.S. Affiliated Pacific Islands. (DOCX)

S2 Table Ebsco/CINAHL search strategy for studies related to pregnancy and perinatal health outcomes among Pacific Islander women in the United States and U.S. Affiliated Pacific Islands. (DOCX)

S3 Table Ovid/PsycINFO search strategy for studies related to pregnancy and perinatal health outcomes among Pacific Islander women in the United States and U.S. Affiliated Pacific Islands. (DOCX)

S4 Table Ovid/EMBASE search strategy for studies related to pregnancy and perinatal health outcomes among Pacific Islander women in the United States and U.S. Affiliated Pacific Islands. (DOCX)
