# Supplementary material for: Pregnancy health and perinatal outcomes among Pacific Islander women in the United States and US Affiliated Pacific Islands: Protocol for a scoping review
Source: PLoS One. 2022 Jan 18;17(1):e0262010. doi: 10.1371/journal.pone.0262010 (PMC8765672; doi:10.1371/journal.pone.0262010)
Supplement: S1 Table — (DOCX) [file pone.0262010.s002.docx]

**S1 Table. Ovid/MEDLINE search strategy for studies related to pregnancy and perinatal health outcomes among Pacific Islander women in the United States and U.S. Affiliated Pacific Islands.***

| Line | Query | Results |
| --- | --- | --- |
| 1 | exp Oceanic Ancestry Group/ | 10248 |
| 2 | (pacific islander* or (native* adj2 hawai*) or Hawaii or Hawai'i or hawai* or ni'ihau or niihau or "kaua'i" or kauai or "o'ahu" or oahu or "moloka'i" or molokai or "lana'i" or lanai or "kaho'olawe" or kahoolawe or maui or austral islands or Tupua'i islands or Bass islands or australasia* or Caroline Island* or Carolines or carolinian* or chamorro* or chuuk or chuukese or Cook island* or Easter island* or Fiji or Fijian* or Futuna or Guam or Guamanian* or "i-kiribati" or kiribati* or kosrae or kosraean* or maori* or mariana island* or Marianas or Marshall island* or Marshalls or Marshallese or Melanesia* or Micronesia* or New Caledonia* or Niue or Niuean* or Ni-Vanuatu or Vanuatu or pacific island* or palau or palauan* or papua new guinea* or phoenix island* or pitcairn island* or pohnpei or pohnpeian* or polynesia* or rapa nui or saipan* or american samoa* or samoa* or pacific women).mp. | 44499 |
| 3 | exp Pregnancy/ or Pregnant Women/ or maternal-fetal relations/ or exp pregnancy complications/ or exp maternal health services/ or maternal health/ or maternal welfare/ or mothers/ or exp congenital abnormalities/ or exp infant, low birth weight/ or exp infant, premature/ or exp cesarean section/ | 1532554 |
| 4 | (perinatal$ or peri-natal$ or prenatal$ or pre-natal$ or antenatal$ or ante-natal$ or pregnan$ or trimester$ or mother$ or maternal$).mp. | 1268346 |
| 5 | (gestational diabetes or (infection* adj10 pregnan*) or anemia or blood pressure or ((hypertension or hypertensive) adj2 (pregnancy or gestational or maternal)) or hyperemesis or preeclamsia or pre-eclampsia or (medically assisted adj1 (birth* or deliver*)) or maternal mortality or postpartum hemorrhage* or birth defect* or birth weight or fetal macrosomia or ((preterm or premature or pre-term) adj1 (labor or deliver*)) or NICU or stillbirth or neonatal mortality or infant mortality).mp. | 864670 |
| 6 | exp geographic locations/ | 4305096 |
| 7 | cities/ or islands/ or "oceans and seas"/ or exp united states/ or puerto rico/ or american samoa/ or guam/ or (northern mariana islands or federated states of micronesia or marshall islands or USAPI or united states).mp. | 1504418 |
| 8 | ((1 or 2) and (3 or 4 or 5)) not (6 not 7) | 2180 |
| 9 | limit 8 to yr="2010 -Current" | 961 |
| 10 | limit 9 to english language | 956 |

*Searched on August 6, 2020.
