# Supplementary material for: Pregnancy health and perinatal outcomes among Pacific Islander women in the United States and US Affiliated Pacific Islands: Protocol for a scoping review
Source: PLoS One. 2022 Jan 18;17(1):e0262010. doi: 10.1371/journal.pone.0262010 (PMC8765672; doi:10.1371/journal.pone.0262010)
Supplement: S2 Table — (DOCX) [file pone.0262010.s003.docx]

**S2 Table. Ebsco/CINAHL search strategy for studies related to pregnancy and perinatal health outcomes among Pacific Islander women in the United States and U.S. Affiliated Pacific Islands.***

| Limiters - Published Date: 20100101-20201231; English Language  Expanders - Also search within the full text of the articles; Apply equivalent subjects  Search modes - Boolean/Phrase |
| --- |
| (MH (Maori) OR TI (pacific-islander* or (native* N2 hawai*) or Hawaii or Hawai'i or hawai* or ni'ihau or niihau or "kaua'i" or kauai or "o'ahu" or oahu or "moloka'i" or molokai or "lana'i" or lanai or "kaho'olawe" or kahoolawe or maui or austral-islands or Tupua'i islands or Bass-island* or australasia* or Caroline-Island* or Carolines or carolinian* or chamorro* or chuuk or chuukese or Cook-island* or Easter-island* or Fiji or Fijian* or Futuna or Guam or Guamanian* or "i-kiribati" or kiribati* or kosrae or kosraean* or maori* or mariana-island* or Marianas or Marshall-island* or Marshalls or Marshallese or Melanesia* or Micronesia* or New-Caledonia* or Niue or Niuean* or Ni-Vanuatu or Vanuatu or pacific-island* or palau or palauan* or papua-new-guinea* or phoenix-island* or pitcairn-island* or pohnpei or pohnpeian* or polynesia* or rapa-nui or saipan* or American-samoa* or samoa* or pacific-women) or AB (pacific-islander* or (native* N2 hawai*) or Hawaii or Hawai'i or hawai* or ni'ihau or niihau or "kaua'i" or kauai or "o'ahu" or oahu or "moloka'i" or molokai or "lana'i" or lanai or "kaho'olawe" or kahoolawe or maui or austral-islands or Tupua'i islands or Bass-island* or australasia* or Caroline-Island* or Carolines or carolinian* or chamorro* or chuuk or chuukese or Cook-island* or Easter-island* or Fiji or Fijian* or Futuna or Guam or Guamanian* or "i-kiribati" or kiribati* or kosrae or kosraean* or maori* or mariana-island* or Marianas or Marshall-island* or Marshalls or Marshallese or Melanesia* or Micronesia* or New-Caledonia* or Niue or Niuean* or Ni-Vanuatu or Vanuatu or pacific-island* or palau or palauan* or papua-new-guinea* or phoenix-island* or pitcairn-island* or pohnpei or pohnpeian* or polynesia* or rapa-nui or saipan* or American-samoa* or samoa* or pacific-women)) AND (MH (Pregnancy+ OR pregnancy complications+ OR maternal health services+ OR mothers+ OR abnormalities+ OR infant, low birth weight+ OR expectant mothers OR mother-infant relations OR maternal-child health OR maternal welfare OR maternal-child welfare OR cesarean section+ OR maternal mortality OR infant mortality) OR TI (perinatal$ or peri-natal$ or prenatal$ or pre-natal$ or antenatal$ or ante-natal$ or pregnan$ or trimester$ or mother$ or maternal$ or gestational-diabetes or (infection* N10 pregnan*) or anemia or blood-pressure or ((hypertension or hypertensive) N2 (pregnancy or gestational or maternal)) or hyperemesis or preeclamsia or pre-eclampsia or (medically-assisted N1 (birth* or deliver*)) or maternal-mortality or postpartum-hemorrhage* or birth-defect* or birth-weight or fetal-macrosomia or ((preterm or premature or pre-term) N1 (labor or deliver*)) or NICU or stillbirth or neonatal-mortality or infant-mortality) OR AB (perinatal$ or peri-natal$ or prenatal$ or pre-natal$ or antenatal$ or ante-natal$ or pregnan$ or trimester$ or mother$ or maternal$ or gestational-diabetes or (infection* N10 pregnan*) or anemia or blood-pressure or ((hypertension or hypertensive) N2 (pregnancy or gestational or maternal)) or hyperemesis or preeclamsia or pre-eclampsia or (medically-assisted N1 (birth* or deliver*)) or maternal-mortality or postpartum-hemorrhage* or birth-defect* or birth-weight or fetal-macrosomia or ((preterm or premature or pre-term) N1 (labor or deliver*)) or NICU or stillbirth or neonatal-mortality or infant-mortality)) |
| 900 results |

*Searched on August 6, 2020.
